# Developing a sustainable child and family service system after a community tragedy: Lessons from Sandy Hook

**DOI:** 10.1002/jcop.21890

**Published:** 2017-04-21

**Authors:** Kimberly Eaton Hoagwood, Su‐chin Serene Olin, Nicole M. Wang, Michele Pollock, Mary Acri, Elizabeth Glaeser, Emma D. Whitmyre, Amy Storfer‐Isser, Sarah McCue Horwitz

**Affiliations:** ^1^ NYU Langone Medical Center

## Abstract

This article describes a systematic approach to assessing community services post‐Sandy Hook shooting. An evaluation team was invited to develop a sustainability plan for community services in Newtown. Service organizations, providers, and families were interviewed. Descriptive statistics were used to characterize the range of services; respondent perspectives were coded using content analysis. We found that Newtown has a broad array of community services, but respondent groups varied in their perceptions of service adequacy. Consensus existed about core components of an ideal service system, including centralizing access; coordinating care, personalizing and tailoring services for families, and providing evidence‐based care. The strategic community assessment approach developed here may inform how communities examine their service capacity and develop sustainability plans post‐disaster.

Data from the FBI confirms that between 2000 and 2013 mass shootings rose dramatically: there were 115 active‐shooter incidents between 2008 and 2013, compared to 45 from 2000 to 2007 (Blair & Schweit, 2014). The effects of these shootings on communities are profound and include high rates of posttraumatic stress disorder (PTSD), depression, anxiety, and behavioral problems (Escobar, Canino, Rubio‐Stipec, & Bravo, 1992; Foa, Stein, & McFarlane, 2006; Instiute of Medicine, 2003; North, Kawasaki, Spitznagel, & Hong, 2004; Solomon & Green, 1992). Importantly, the effects from these mass shootings often last for years (Bromet, Havenaar, Bromet, & Havenaar, 2009; Norris, Friedman, & Watson, 2002; Ursano, Fullerton, & Benedek, 2009).

A number of evidence‐based interventions designed to target postdisaster symptoms among children and families have been developed to assist in recovery, although most of these target trauma symptoms consequent to events other than shootings (Cohen, Deblinger, Mannarino, & Steer, 2004; Kataoka et al., 2003; Stein et al., 2003). Trainings to support delivery of evidence‐based treatments at scale (i.e., community or statewide) have also been studied (Hoagwood et al., 2007). Even so, community‐based mental health services tend to be significantly underused by those most in need (Wang et al., 2007, 2008).

As a consequence of the increase in mass shootings and other disasters, disaster planning in communities has expanded, and there are many toolkits, trainings, and technical assistance supports available (American Academy of Pediatrics, 2012; American Red Cross, 2016, Federal Emergency Management Agency, 2016; Brymer et al., 2006; Palinkas, 2012, 2015; Shannon, 2015). However, these programs and supportive materials largely focus on identifying immediate and short‐term community needs, training and deploying appropriate staff after a disaster, and safety issues. These efforts do not address service system issues, including the need to buttress existing services, construct a new and higher quality system responsive to the demands of the new healthcare environment, or address the long‐term needs of communities that have experienced these tragedies. In fact, little is known about rebuilding or redesigning sustainable service systems for children and families after a tragedy.

In January of 2015, The Office for Victims of Crime (OVC), a component of the Office of Justice Programs, U.S. Department of Justice, contacted the New York University Center for Implementation‐Dissemination of Evidence‐based Practices Among States (The IDEAS Center), requesting that we work with the Town of Newtown, the site of the Sandy Hook Elementary School shooting, to assist the community in developing a plan to redesign and sustain their services for the future. The Sandy Hook shooting tragedy occurred on December 14, 2012, when Adam Lanza, a 20‐year old resident of Newtown, CT, went to Sandy Hook Elementary School and fatally shot and killed 20 elementary school students and six staff members; two others were wounded. In addition to the families of the deceased, victims included 12 first‐grade students who survived the shootings; approximately 500 more Sandy Hook Elementary School students, teachers, and staff; and traumatized police officers and first responders. The effect of the tragedy led to OVC declaring the entire town of approximately 27,560 residents the victim.

During the 3 years after this event, the Town of Newtown, like other communities where mass tragedies have occurred, received both public (e.g., federal, state, and local) and private funding to meet the needs of its citizens. The Town of Newtown distributed some of these public and private resources, and other resources went directly to local community organizations, the school district, and local mental health service providers. Anticipating the need for a sustainability plan once federal funding ended, the OVC asked NYU researchers to assist. Specifically, we were asked to (a) describe the type and organization of services that currently exist; (b) gather perspectives from both the services providers and the families affected by the tragedy on the adequacy of existing services; and (c) make recommendations, based on the data generated, for improving and sustaining high‐quality, integrated services.

This article summarizes our approach, our findings, and our recommendations. The approach we took may be useful in assisting other communities to systematically assess the architecture of their system and to strengthen and sustain high‐quality services. The institutional review board evaluated this project and deemed it a quality improvement effort because of its strong focus on local assessment to improve service quality.

## 1 METHOD

### 1.1 Study samples

To gather the data necessary to answer the questions posed by OVC, we interviewed community service organizations, private behavioral health providers, and both victim and witness families.

#### 1.1.1 Organizations

In collaboration with town leadership and local stakeholders, the research team developed a list of all local organizations to interview, including direct service providers, medical providers, community‐based organizations, faith‐based organizations, and representatives from local government. The First Selectman (the title for the town's mayor) sent an introductory letter to leadership at all identified organizations. The research team then followed up by phone and e‐mail to provide additional information about the project, answer questions, and schedule interviews. Because we were interested in the agency perspective and prior research suggests that no one individual has the complete organizational perspective, we conduced group interviews when agencies requested that multiple staff be interviewed. A total of 30 organizations were invited to participate in interviews (see Table 1). Group interviews were conducted with 56 individuals from 28 organizations (the number of participants per group interview ranged from one to six, with an average of two per organization). Leadership at two organizations (both medical providers) did not return our calls and emails.

**Table 1:** Participants by Type of Organization

	No. organizations invited	No. organizations interviewed	Individuals per group interview	
	N=30	N=28	Mean	Range
Behavioral health	**7**	**7**	3.14	1–6
Medical	7	5	1.4	1–3
Police	1	1	4.00	n/a
School district	1	1	6.00	n/a
Faith‐based orgs	5	5	1.00	1–1
Local government	4	4	1.00	1–1
Foundations/other CBOs	5	5	1.60	1–2

*Note*. CBO = Community‐based organization.

#### 1.1.2 Private providers

A list of all 57 behavioral health and private mental health practitioners within a 5‐mile radius of Newtown, CT, was generated. To ensure an adequate representation of the opinions of the private mental health providers serving the Newtown community, we estimated that it was necessary to interview 25% of the 57 individuals identifiable through public records. Given that we wanted this sample to be unbiased and that we had no information on a probable response rate, we randomly selected samples of providers, contacted them to arrange telephone or in‐person interviews, and then randomly selected additional samples from the provider list until we approached our target sample of 14 interviews. The First Selectman sent the same introductory letter to all selected private providers. Of the 39 randomly selected individual providers we could reach (two could not be contacted), 14, or 35.9%, agreed to participate and were interviewed. Like the entire sample of 57 providers, the 14 interviewed were largely master's‐level licensed clinical social workers (LCSWs) (35.7%) or other master's‐level clinicians (35.7%). However, clinicians from other disciplinary backgrounds (e.g., PsyD, PhD) were overrepresented (28.6% vs. less than 15% in the full sample) in the group we interviewed.

#### 1.1.3 Families

A total of 28 families were invited to participate in interviews (17 victim families, 11 witness families). Although there were 26 victim families (i.e., families whose children died) and 12 witness families (i.e., families whose children directly witnessed the shooting), we included only those families who currently live in Newtown. The First Selectman sent an e‐mail to the victim families that provided basic project information and the research team's direct contact information, for anyone interested in participating. The research team also sent the same letter to these families through their monitored media request website. The First Selectman and the Executive Director of the Newtown‐Sandy Hook Community Foundation sent an e‐mail to the witness families that provided basic project information and the research team's direct contact information, for anyone interested in participating. Families were contacted only once and the research team never directly contacted a family prior to the family contacting the team. Of the 28 contacted families, 10 (58.8%) victim families and 4 (36.4%) witness families chose to participate.

### 1.2 Measures

Before the scheduled interview, organizations and private practitioners were sent a questionnaire asking a range of questions about their client population, organizational structure, sources of revenue, types of services offered, types of treatments offered, and staff training opportunities. Questions were adapted from ongoing research with New York State behavioral health agencies (Chor et al., 2014; Chor, Wisdom, Olin, Hoagwood, & Horwitz, 2015; Olin et al., 2015a,b). The in‐person interviews asked about barriers and facilitators to implementing and sustaining services, current and future needs of the community, local professional networks, and characteristics of an ideal services system.

The family interview asked seven open‐ended questions on services families had received, match of services to needs, most and least helpful services, services needed but not received, and what would be an ideal set of services. Responses were recorded and questions were followed‐up with structured probes when necessary.

### 1.3 Coding of family responses

To develop codes for the open‐ended questions in the family interviews, two of the authors (SSO and SMH) first used qualitative content analysis techniques (Hsieh & Shannon, 2005), using two randomly selected family interviews. These codes were then used to code two additional, randomly selected family interviews with complete agreement. The project staff coded the remaining interviews (10), after training, with 30% being recoded to check for accuracy. Any discrepancies were reconciled through a discussion between coders.

### 1.4 Analysis

Categorical measures were summarized using counts and percentages, while continuous measures were described using means and standard deviations. Univariate descriptive statistics are shown for the 14 solo providers and the seven groups of community‐based providers (behavioral health [n = 7], medical [n = 5], police [n = 1], school [n = 1], faith‐based organizations [n = 5], local government [n = 4], and foundations/other community‐based organizations [n = 5]). Counts and percentages were used to summarize bivariate associations between the primary revenue source and type of treatment offered (evidence‐based and nonevidence based). Data were analyzed using SAS (version 9.3).

## 2 RESULTS

### 2.1 Demographic characteristics of the community providers and services

We interviewed 56 individuals across the seven groups of community organizations as well as 14 solo practitioners. Most providers had advanced degrees (i.e., a master's degree or more), and, with the exception of the police, clergy and foundations/other community‐based organizations, most of the individuals interviewed were female. While the majority had been in their current position 5 years or less, they had been in their current fields 10 years or more. The single most common certification was that of an LCSW. The solo practitioners’ characteristics were similar to those of the organization interviewees. Among solo practitioners, five were LCSWs, five were licensed professional counselors or licensed marriage and family therapists, and four had “other” clinical licensure. Organizations that employed staff ranged from a low of 2–20 staff to a high of ≥ 100 staff, with most providing direct services. The majority of behavioral health care organizations anticipated reduced staffing (57%) after December of 2015.

We looked at the degree to which services were tailored or personalized to meet victim needs (Table 2). Surprisingly, few of the 12 specific mental health services were tailored to meet victim needs. For example, among the seven community‐based organizations providing behavioral health services, only 3 of 12 services (i.e., family therapy, home‐based family therapy, and crisis services) were reported by at least 50% of agencies delivering these services to be tailored for victims. A similar lack of tailoring services for victims was reported by these agencies for specific health, substance abuse treatment, and social services (data not shown). Similar patterns were observed among the solo practitioners. Further, only 1 of 28 organizations mentioned offering bereavement or grief counseling to families. This absence of tailored services for victim's families was also observed for the wellness, recreation, and financial services (data not shown).

**Table 2:** Specific Services Provided by Type of Organization (N=42 Community‐Based Providers; n=28 Organizations, n=14 Solo Practitioners)

	Behavioral health (n=7)	Medical (n=5)	Police (n=1)	School district (n=1)	Faith‐based organizations (n=5)	Local government (n=4)	Foundations other CBOs (n=5)	Solo practice (n=14)^a^
**Mental health services tailored for victims**								
Family support services	2/6 (33.3)	3/4 (75.0)	n/a	1/1 (100)	0/2 (0)	1/1 (100)	n/a	2/5 (40.0)
Family therapy	3/4 (75.0)	1/2 (50.0)	n/a	n/a	n/a	0/1 (0)	n/a	3/9 (33.3)
Group therapy	1/4 (25.0)	1/2 (50.0)	n/a	1/1 (100)	0/1 (0)	0/1 (0)	n/a	2/5 (40.0)
Home‐based / family preservation	1/2 (50.0)	n/a	n/a	n/a	n/a	n/a	n/a	1/2 (50.0)
School‐based MH services	0 (0)	1/1 (100)	n/a	1/1 (100)	0/1 (0)	n/a	n/a	n/a
Individual child therapy	2/5 (40.0)	1/2 (50.0)	n/a	1/1 (100)	0/1 (0)	n/a	n/a	5/9 (55.6)
Individual adult therapy	0/3 (0)	n/a	n/a	n/a	n/a	0/1 (0)	n/a	7/11 (63.6)
Case management	0/3 (0)	n/a	n/a	n/a	n/a	0/1 (0)	n/a	n/a
Psychotropic medication	1/3 (33.3)	0/2 (0)	n/a	n/a	n/a	0/1 (0)	n/a	0/1 (0)
Crisis services	2/3 (66.7)	1/2 (50.0)	n/a	n/a	2/3 (66.7)	0/1 (0)	n/a	3/4 (75.0)
Partial day treatment	0/1 (0)	0/1 (0)	n/a	n/a	n/a	n/a	n/a	n/a
Other MH services	n/a	1/1 (100)	n/a	n/a	1/2 (50.0)	n/a	n/a	n/a
**Offer any EB treatments**	4 (57.1)	2 (40.0)	0 (0)	1 (100)	0 (0)	0 (0)	0 (0)	10 (83.3)
Offer any treatments with weak evidence	5 (71.4)	1 (20.0)	0 (0)	1 (100)	2 (40.0)	0 (0)	1 (20.0)	5 (41.7)
Offer any treatments with no evidence	5 (71.4)	0 (0)	0 (0)	0 (0)	1 (20.0)	0 (0)	0 (0)	6 (50.0)

*Note*. CBO = Community‐based organization; MH = Mental health; EB = Evidence‐based.

Evidence‐based treatments include psychotropic medication management, eye movement desensitization and reprocessing, cognitive behavioral therapy, trauma‐focused cognitive behavioral therapy, cognitive behavioral intervention for trauma in schools, child‐parent psychotherapy, cognitive processing therapy, and child and family traumatic stress intervention. Treatments with weak evidence include biofeedback/neurofeedback, animal‐assisted therapy, emotional freedom technique / tapping, acupuncture, support groups, parenting groups, reiki, massage therapy, and meditation/yoga.

Treatments with no evidence include wrap‐around services, trauma systems therapy, somatic experiencing, brainspotting, music therapy, art or other expressive therapies, play therapy, aromatherapy, internal family systems, and MNRI®.

^a^These data are available for 12 of the 14 solo practitioners.

We next inquired about the availability of treatments that have been examined empirically and found to have a strong evidence base (evidence‐based practices or EBPs) for the treatment of trauma, stress, anxiety, or depression (Table 2). Only four of the seven behavioral health organizations (i.e., 57%) and two of the medical organizations (i.e., 40%) offered evidence‐based trauma treatments. In contrast, the school system and the solo practitioners (83%) offered EBPs. When we examined treatments for trauma with a limited or weak evidence base, we found that five of seven (71%) behavioral health organizations, five other organizations, and 42% of solo practitioners also offered treatments with a weak evidence base. We also examined the availability of services with no evidence base and found that five of seven (71%) behavioral health agencies and half of the solo practitioners offered services with no evidence base.

### 2.2 What does Newtown need to create an ideal service system?

We asked respondents about the characteristics they believed were needed to construct an ideal service system in their community (Table 3). The two areas provider organizations cited most frequently were (a) developing centralized community supports and (b) improving collaboration/coordination among community providers. The solo practitioners also endorsed a wide range of strategies including centralized community support, education and training, collaboration/coordination among different service providers, and the creation of an integrated care service with a continuum of services (Table 3).

**Table 3:** Characteristics of and Ingredients to Develop a Service System That Would Be Responsive to the Needs of Your Community by Type of Organization (N=42 Community‐Based Providers; n=28 Organizations, n=14 Solo Practitioners)

	Behavioral health (n=7)	Medical (n=5)	Police (n=1)	School district (n=1)	Faith‐based (n=5)	Local government (n=4)	Foundations/other CBOs (n=5)	Solo practice (n=14)
**Community factors**								
Decision maker / leadership	1 (14.3)	2 (40.0)	0 (0)	0 (0)	0 (0)	1 (25.0)	0 (0)	0 (0)
Expert consultant (trauma services)	2 (28.6)	0 (0)	1 (100)	1 (100)	0 (0)	0 (0)	1 (20.0)	1 (7.1)
Independent consultant / arbitrator	1 (14.3)	0 (0)	0 (0)	0 (0)	0 (0)	1 (25.0)	0 (0)	1 (7.1)
Advocate for victims	1 (14.3)	1 (20.0)	0 (0)	0 (0)	0 (0)	0 (0)	0 (0)	0 (0)
Community building activities	1 (14.3)	1 (20.0)	0 (0)	0 (0)	0 (0)	0 (0)	0 (0)	2 (14.3)
Communication/dissemination of information	1 (14.3)	1 (20.0)	0 (0)	0 (0)	1 (20.0)	1 (25.0)	1 (20.0)	1 (7.1)
Centralized community support	3 (42.9)	2 (40.0)	1 (100)	0 (0)	3 (60.0)	2 (50.0)	2 (40.0)	5 (35.7)
Education / training and supports	1 (14.3)	0 (0)	0 (0)	0 (0)	1 (20.0)	0 (0)	1 (20.0)	5 (35.7)
Providing a voice for stakeholders	2 (28.6)	0 (0)	0 (0)	0 (0)	1 (20.0)	0 (0)	1 (20.0)	1 (7.1)
Other	0 (0)	1 (20.0)	0 (0)	0 (0)	3 (60.0)	0 (0)	1 (20.0)	4 (28.6)
**Organizational factors**								
Continued needs assessment /identify needs	0 (0)	0 (0)	1 (100)	0 (0)	1 (20.0)	0 (0)	0 (0)	2 (14.3)
Collaboration/coordination among providers	3 (42.9)	3 (60.0)	1 (100)	1 (100)	1 (20.0)	1 (25.0)	1 (20.0)	4 (28.6)
Creation of a one‐stop shop with a complete continuum of services	1 (14.3)	1 (20.0)	0 (0)	0 (0)	0 (0)	2 (50.0)	1 (20.0)	4 (28.6)
Clarity about mission	1 (14.3)	1 (20.0)	0 (0)	0 (0)	0 (0)	1 (25.0)	0 (0)	0 (0)
Other	2 (28.6)	1 (20.0)	0 (0)	1 (100)	0 (0)	0 (0)	0 (0)	4 (28.6)
**Individual factors**								
Access to services	0 (0)	0 (0)	1 (100)	0 (0)	0 (0)	0 (0)	1 (20.0)	2 (14.3)
Care navigator	1 (14.3)	1 (20.0)	0 (0)	0 (0)	1 (20.0)	1 (25.0)	0 (0)	0 (0)
**Role of health or medical providers**								
Collaboration among MH service providers	1 (14.3)	0 (0)	1 (100)	0 (0)	1 (20.0)	0 (0)	1 (20.0)	1 (7.1)
Clarity of different agency missions	0 (0)	0 (0)	1 (100)	0 (0)	0 (0)	0 (0)	0 (0)	1 (7.1)
Develop competency in identification of trauma related needs	1 (14.3)	0 (0)	0 (0)	0 (0)	1 (20.0)	0 (0)	0 (0)	5 (35.7)
Other	2 (28.6)	2 (40.0)	0 (0)	0 (0)	3 (60.0)	1 (25.0)	2 (40.0)	5 (35.7)
**Necessary to make the ideal system a reality**								
Town support	2 (28.6)	0 (0)	0 (0)	0 (0)	0 (0)	0 (0)	1 (20.0)	0 (0)
Funding / transparency about money	4 (57.1)	4 (80.0)	0 (0)	0 (0)	2 (40.0)	2 (50.0)	0 (0)	5 (35.7)
Leadership / decision maker	3 (28.6)	0 (0)	0 (0)	1 (100)	2 (40.0)	0 (0)	0 (0)	1 (7.1)
Trust	0 (0)	1 (20.0)	1 (100)	1 (100)	1 (20.0)	0 (0)	0 (0)	0 (0)
Willingness to set aside differences	3 (42.9)	0 (0)	0 (0)	0 (0)	0 (0)	0 (0)	2 (40.0)	2 (14.3)
Communication / dialog for stakeholders	3 (42.9)	3 (60.0)	0 (0)	0 (0)	0 (0)	2 (50.0)	1 (20.0)	1 (7.1)
Coordination of services through single point of contact	2 (28.6)	1 (20.0)	1 (100)	0 (0)	0 (0)	1 (25.0)	0 (0)	5 (35.7)
Clarity of different agency missions	1 (14.3)	1 (20.0)	0 (0)	0 (0)	0 (0)	0 (0)	0 (0)	0 (0)
Need for school‐based services	0 (0)	1 (20.0)	0 (0)	0 (0)	0 (0)	1 (25.0)	0 (0)	3 (21.4)
Other	1 (14.3)	1 (20.0)	0 (0)	0 (0)	3 (60.0)	1 (25.0)	1 (20.0)	7 (50.0)

*Note*. CBO = Community‐based organization.

Finally, we asked the provider organizations about the necessary ingredients to make the ideal service system a reality (Table 3). Both organizations and solo practitioners felt funding and transparency about money was important. Organizations also thought communication or dialogue among stakeholders, leadership or a decision maker, willingness to set aside differences, and communication and coordination of services through a *single* point of entry were important. Solo practitioners thought development of competency in identifying trauma‐related needs and coordinated care with a single point of entry were important (Table 3).

### 2.3 Association of chief sources of funding with use of EBPs

When we examined the association of type of funding with whether organizations offered evidence‐based mental health treatments, we found that three of five (or 60%) behavioral health organizations reported offering EBPs if they received federal/state/government (F/S/G) funding, but four of five (or 80%) also offered treatments with weak or no evidence base (Table 4). The one behavioral health organization with charitable funds as a major source of funding offered only treatments with weak or no evidence base. The one medical provider whose primary source of revenue was F/S/G offered no EBPs, while, of the other four medical providers relying on other sources of income (private and other), 50% offered some EBPs. In contrast, 83.3% of solo practitioners, all of whom relied on direct payments or insurance, offered EBPs. Two thirds (4/6; 67%) of organizations that relied on F/S/G funding reported use of treatments with weak or no evidence base, while less than half (8/18; 44%) of those who rely primarily on other direct payments or insurance used these treatments.

**Table 4:** Association of Primary Source of Revenue With Types of Treatments Offered by Organization Type Among Organizations Likely to Provide Evidence‐Based Treatments

	Treatments are evidence‐based		Treatments that are not evidence‐based	
	No	Yes	No	Yes
Behavioral health (n=7)				
Federal/state/government (OVC) funds (n=5)	2 (40.0)	3 (60.0)	1 (20.0)	4 (80.0)
Private charitable funds (n=1)	1 (100)	0 (0)	0 (0)	1 (100)
Other (insurance, direct pay, no answer) (n=1)	0 (0)	1 (100)	0 (0)	1 (100)
Medical (n=5)				
Federal/state/government (OVC) funds (n=1)	1 (100)	0 (0)	1 (100)	0 (0)
Private charitable funds (n=1)	0 (0)	1 (100)	0 (0)	1 (100)
Other (insurance, direct pay, no answer) (n=3)	2 (66.7)	1 (33.3)	3 (100)	0 (0)
School district (n=1)				
Town funds (n=1)	0 (0)	1 (100)	0 (0)	1 (100)
Individual practice (n=14)				
Other (insurance, direct pay, no answer; (n=14)^a^	2 (16.7)	10 (83.3)	5 (41.7)	7 (58.3)

*Note*. OVC = Office for Victims Crime.

^a^Information provided for 12 of 14 providers.

### 2.4 Victim family and witness family perspectives

Table 5 displays the data on the mental health services received and the perceived benefit from these services for both victim and witness families. All but one of the victim families interviewed, and half of the witness families interviewed, received traditional mental health services locally. Victim families (20%) infrequently received navigator services, whereas all witness families interviewed (100%) received such services. Victim families frequently reported receiving money to pay for services (70%). Families were not always clear about the sources of these funds.

**Table 5:** Services Received and Perceived Benefit of Services by Victim and Witness Families

	Victim families (n=10)	Witness families (n=4)
**Services received**		
Local community services		
Traditional MH services	9 (90.0)	2 (50.0)
Trauma/grief‐specific	2 (20.0)	0 (0)
Unspecified	7 (70.0)	2 (50.0)
Alternative or new therapies (e.g., MNRI®, tapping)	2 (20.0)	2 (50.0)
My Sandy Hook Family Fund (volunteer program)	2 (20.0)	0(0)
Navigator to help with referrals/care coordination/insurance	2 (20.0)	4 (100)
Money / allowance for services	7 (70.0)	2 (50.0)
Nonlocal community services		
Traditional MH services	2 (20.0)	4 (100)
Trauma/grief‐specific	2 (20.0)	4 (100)
Alternative or new therapies (e.g., MNRI®, tapping)	0 (0)	0 (0)
CT state trooper (safety, security)	2 (20.0)	0 (0)
OVC/OVS case worker / liaison	3 (30.0)	0 (0)
Other	1 (10.0)	0 (0)
School‐based services		
School family liaison	3 (30.0)	0 (0)
School‐based MH services	0 (0)	1 (25.0)
Academic supports (including tutoring)	1 (10.0)	1 (25.0)
Other (e.g., Yale, NCTSN)	0 (0)	3 (75.0)
Overall, how well did the services match your needs		
Not well	5 (50.0)	1 (25.0)
Somewhat well	0 (0)	1 (25.0)
Very well	2 (20.0)	2 (50.0)
Varied	2 (20.0)	0 (0)
No answer	1 (10.0)	0 (0)
Any services that were needed but were not provided		
No	3 (30.0)	1 (25.0)
Yes	5 (50.0)	3 (75.0)
Don't know	2 (20.0)	0 (0)
**Three most helpful services**	22 responses from 8 families	11 responses from 4 families
Mental health	7	6
Alternative or new therapies	4	0
Volunteer	2	0
RRT	0	2
OVC/OVS case worker / liaison / entitlements assistance	3	0
State troopers	2	0
School‐based	4	1
Financial	0	1
Community‐based organizations	0	1
**What made the services helpful**		
Point person	4 (40.0)	3 (75.0)
Referral services	0 (0)	1 (25.0)
Money/entitlements for services	4 (40.0)	2 (50.0)
Trauma‐specific services	2 (20.0)	3 (75.0)
Availability / intensity / timing of services	3 (30.0)	1 (25.0)
Addressed a specific needs (e.g., tutoring)	5 (50.0)	3 (75.0)
Other reason why services were helpful	0 (0)	1 (25.0)
**Three least helpful services**	10 responses from 7 families	4 responses from 2 families
Mental health	2	2
Alternative or new therapies	1	1
RRT	3	0
OVC/OVS case worker / liaison	2	0
School‐based	1	1
Financial	1	0
**What made the services unhelpful**		
Difficulty getting reimbursement	4 (40.0)	1 (25.0)
Provider expertise did not match need	4 (40.0)	2 (50.0)
Service type/intensity/timing did not match need	6 (60.0)	2 (50.0)
Other	1 (10.0)	0 (0)
Not sure	3 (30.0)	1 (25.0)
**Benefit from services**		
Rank (scale from 1=not at all to 10=a great deal)		
Overall (mean ± SD)	4.9 ± 3.2	8.3 ± 2.1
Number rated below average (rank 1‐4)	3 (30.0)	0 (0)
Number rated neutral (rank 5)	4 (40.0)	0 (0)
Number rated above average (rank 6‐10)	3 (30.0)	4 (100)
Reason for high rating on benefit from services (can select multiple answers)		
Addressed specific need	0 (0)	1 (25.0)
Provider expertise matched need	0 (0)	0 (0)
Service type/timing/intensity matched need	1 (10.0)	1 (25.0)
Person receiving services got better	0 (10.0)	2 (50.0)
No answer	2 (20.0)	2 (50.0)

*Note*. OVC = Office for Victims of Crime; SD = standard deviation; OVS = Office of Victim Services; MNRI® = Masgutova Neurosensorimotor Reflex Integration®; NCTSN = National Child Traumatic Stress Network.

Overall, 50% of the victim families reported that the services they received were not well matched to their needs. Only 20% of the victim families gave a “very well” rating when asked how well the services matched their needs. Among the witness families interviewed, half rated their services as having matched their needs very well. Both sets of families reported that they needed services that they did not receive (50% victim families, 75% witness families; Table 5).

There was a range of features that distinguished helpful services. For victim families, services were helpful when they addressed a specific need; in addition, they found having a point person and money/entitlements for services helpful. For witness families, having a point person, services that were trauma specific, and addressing a specific need were the most important features.

The characteristics of services that made them least helpful for victim families included the service itself not matching the family's needs, difficulties with reimbursement, and mismatch between provider expertise and family need. Service and provider expertise mismatches were the most frequently mentioned characteristic by witness families interviewed. On a scale ranging from 1 (*not at all*) to 10 (*a great deal*), victim families rated the overall benefit from services as 4.3 ± 3.2, and witness families rated overall benefit as 8.3 ± 2.1. Although the data were not collected in a format that allowed us to test the association between benefit and the specific services received, notes from the interviews suggest that specific evidence‐based trauma‐focused services sought outside of the community were perceived to be most beneficial.

Table 6 displays families’ opinions about the characteristics of an ideal service system. At the community level, victim families thought communication among organizations, including a family voice when designing services, and a mechanism to integrate family voice to oversee use of funds were the most important. At the provider/organization level, victim families felt that additional mental health services and expert trauma services were important. Victim families suggested that that school services be differentiated based on whether the child was a sibling of a victim versus a witness. On the individual level, the three most frequently mentioned features of an ideal system were individualized services for each family, that services be offered to families rather than families having to seek them out, and having a single point of contact. This is consistent with provider perceptions about service access being the greatest current need.

**Table 6:** Recommendations for Ideal Service System Described by Victim and Witness Families

	Victim families (n=10)	Witness families (n=4)
**Ideal service system**		
Community / town		
Effective communication among organizations (government/school district/families)	5 (50.0)	0 (0)
Vetting process for legitimate/appropriate provider/services	2 (20.0)	1 (25.0)
Needs assessment by the town for those directly affected	2 (20.0)	0 (0)
Transparency about funds and tiered allocation	1 (10.0)	0 (0)
Include family voice in service design/how money is spent	4 (40.0)	0 (0)
Other	2 (20.0)	0 (0)
Organizations / providers		
Additional expert trauma services	3 (30.0)	1 (25.0)
Additional local expert trauma services	0 (0)	2 (50.0)
Additional mental health services	4 (40.0)	1 (25.0)
Additional substance abuse services	1 (10.0)	0 (0)
School district		
Differentiated services based on exposure to trauma	3 (30.0)	1 (25.0)
Trauma expertise	0 (0)	1 (25.0)
Academic supports	1 (10.0)	1 (25.0)
Other (e.g., support for teachers)	1 (10.0)	2 (50.0)
Individuals		
Single point of contact for each family	4 (40.0)	2 (50.0)
Need to understand and be able to access entitlements/services	3 (30.0)	1 (25.0)
Services should be offered to victim's families; families should not have to seek out services	6 (60.0)	2 (50.0)
Individualized services for each family	6 (60.0)	3 (75.0)
Privacy protection	3 (30.0)	0 (0)
Fair allocation of funds for trauma‐related services	3 (30.0)	0 (0)

Witness families had few recommendations about an ideal service system at the community level. Their recommendations focused on the provider/organization level, where they also thought that expert trauma services and additional mental health services were important, but added that there should be accessible (i.e., local) expertise to facilitate long‐term continuity of care. They agreed that differentiated services (i.e., for siblings vs. witness children) were needed in schools and also thought that schools needed trauma expertise and academic supports. At the individual level, the important features of an ideal system for witness families included individualized services for each family, a single point of entry, and having services offered to families rather than families having to seek them out on their own.

## 3 DISCUSSION

Events like Sandy Hook are impossible to predict but tragically are occurring with some regularity. Like other mass shootings, the Sandy Hook tragedy affected the entire country because of its prominence in the public eye and media. It is not surprising, therefore, that when the Office for Victims of Crime offered assistance to Newtown after the tragedy, they deemed the entire community “a victim” in order to ensure that funds would flow quickly into the town. This decision, however, likely had consequences for how the system was shaped, what services were provided, and to whom they were provided. It likely also affected how the providers, victim families, and witness families ultimately perceived the services. In addition, before our evaluation, a “division of labor” had been set up between the Department of Education's support for assisting the school system in responding to the tragedy and the Department of Justice's response to assisting the community. This created some challenges. We were asked to focus on the community set of services, not the array of services already being provided in the schools, some of which included community outreach.

Our findings highlighted several aspects of service system redesign that may be relevant to other communities as they assess their capacities for providing sustainable services after a community tragedy. Newtown was unusually well resourced in the aftermath, with funds from federal, state, and private donations. While funding matters, we learned that coordinated and systematic attention to implementation as a separate step in a community's healing is essential. It involves a unique set of tasks, responsibilities, and planning. Developing a communitywide plan for implementation of networked community services needs to involve all major stakeholders sharing a common goal. It involves transparency in leadership, functions, roles, and perhaps funding. As community tragedies may, unfortunately, be expected to continue, communities, states, and national organizations need to think about ways to create stronger and tighter networked services, so there can be more rapid coordinated responses when the need arises. Some guidelines for helping communities create linkages to ensure a more coordinated community response exist (The Office for Victims of Crime, 2015, August).

Currently, several natural experiments are underway as part of Robert Wood Johnson's Culture of Health (Plough et al., 2015) and as part of health systems change (i.e., 3.0 health systems transformation of Halfon et al., 2014). Similarly, Communities that Care initiatives (Hawkins, Oesterle, Brown, Abbott, & Catalano, 2014) are catalyzing neighborhood experiments to infuse evidence‐based services into communities while modifying components to improve implementation. Some of the core components across these natural experiments include centralizing access to services; coordinating care by creating teams that work within and across agencies to provide efficient and nonduplicative care; and personalizing services to improve the match between patient's needs and the care they receive. Interestingly, providers and families in Newtown also identified these themes, being implemented across other U.S. communities, as key features for an improved future system. However, the context of having experienced a tragedy of unprecedented proportions, having to rebuild a sense of community again, and dealing with the wide range of very different personal needs—from those who lost a child to those who were shocked and saddened by the event—brought the challenges of these system issues into stark relief.

Newtown's community service system was broad and included a wide array of different community services—mental health, substance use, medical, social services, schools, faith‐based, and safety‐focused. It was by most standards adequately staffed, with unusually high levels of advanced degrees and years of tenure in the system. The quality of the services, however, was uneven, with significant gaps and unmet needs. Access was largely decentralized with different entry points for school services, community‐based treatment services, and wellness services. Evidence‐based practices were offered in more than half (57%) of the behavioral health agencies, 40% of medical providers, in the school system, and by the majority of the solo providers. Yet services were not individualized, and few personalized or tailored services were available. Surprisingly, only one of the 28 agencies offered bereavement or grief counseling.

There was, however, considerable agreement from providers and families about what services were needed to improve the system. Both providers and families agreed that improving access through centralization of services, improving coordination across agencies, and individualizing services were paramount. There were also suggestions to make monetary decisions about allocation of funds more transparent and provide training on effective organizational practices to all levels of staff: frontline, mid‐management, and leadership.

### 3.1 Limitations

It is important to note the key limitations that may have influenced our findings. Although we were able to interview more than 90% of the provider organizations in Newtown, reaching solo practitioners was much more challenging. Because of the likelihood of self‐selection, our findings from this group of respondents should be interpreted with caution. It is likely that those solo practitioners who agreed to participate were self‐selected based on valuing research. In addition, because we adhered to OVC's classification of the entire town as “victim,” we took a broad approach in understanding services provided to anyone in the town and thus do not have information about which tier of victims these solo practitioners served. Further, while victim families were well represented, witness families were much more reluctant to participate in this evaluation. It is thus likely that the views of the witness families who agreed to participate may not fully represent the range of perspectives from this important group.

### 3.2 Conclusion

The opportunity for an external group of evaluators to assess an entire array of community services within a single town and develop a sustainability plan was very unique. Our approach, which was to sample the entire set of agencies, providers, and families and use semistructured interviews to compare and contrast perspectives from providers and families, yielded data that captured, descriptively, the town's capacities, gaps, and community goals. Despite variability in views about the adequacy of services within the community, there was consensus across providers and families about key components of an ideal service system, including developing an infrastructure for coordination and centralization of services and resources to support all families, but especially those most impacted by the tragedy. Providers and families also recognized the importance of community leadership in sustaining trust and providing a foundation for an ideal service system.

That consensus existed around the elements needed to build a sustainable system is important and bodes well for making these changes happen. That healthcare system leaders have also identified these same components nationally (Berwick, 2016; Halfon et al., 2014; Pronovost & Bo‐Linn, 2012) is noteworthy. National organizations and federal agencies are developing resources and guidelines to inform stronger community policies to deal with tragedies. The core ingredients of community strengthening in the aftermath of a tragedy have been identified and there is now general consensus on these ingredients. Building them a priori into a coordinated, integrated, and sustainable system that is ready for mobilization when tragedy strikes is the next step.
